# Impact of Contextual Factors on the Perceived Participation of People With Multiple Sclerosis and Gait Impairment Using Mobility Assistive Devices: A Qualitative Analysis

**DOI:** 10.1111/hex.70033

**Published:** 2024-09-28

**Authors:** Elise‐Marie Dilger, Nadja Reeck, Dyon Hoekstra, Annett Thiele, Anna Levke Brütt

**Affiliations:** ^1^ Junior Research Group for Rehabilitation Sciences, Department of Health Services Research School of Medicine and Health Sciences, Carl von Ossietzky University of Oldenburg Oldenburg Germany; ^2^ Research Group for Education and Didactics in Chronic and Progressive Diseases and Physical Disabilities, Department of Special Needs Education and Rehabilitation School of Educational and Social Sciences, Carl von Ossietzky University of Oldenburg Oldenburg Germany; ^3^ Department of Medical Psychology University Medical Center Hamburg‐Eppendorf Hamburg Germany

**Keywords:** gait impairment, inclusion, mobility assistive device, multiple sclerosis, participation, qualitative research

## Abstract

**Introduction:**

People with multiple sclerosis and gait impairment are particularly susceptible to facing restrictions in their participation. This study aims to investigate (a) which contextual factors within the framework of the International Classification of Functioning, Disability and Health (ICF) are relevant for participation from the perspective of people with multiple sclerosis and gait impairment who (intend to) use mobility assistive devices, and (b) how these contextual factors shape the subjective facets of participation, including a sense of connection, efficacy and meaning, based on the social identity approach to health and well‐being.

**Methods:**

We conducted a qualitative analysis on data gathered from four online focus group interviews, each involving four to six people with multiple sclerosis and gait impairment who (intend to) use mobility assistive devices (totalling *N* = 19), and 12 individual online interviews conducted with participants from the focus group interviews. The analysis followed the qualitative content analysis according to Kuckartz.

**Results:**

Mobility assistive devices such as walkers, manual wheelchairs and electric wheelchairs/scooters were seen as facilitators that enabled participation in various life situations and enhanced a sense of efficacy by promoting independence. Challenges were encountered in relation to architectural barriers, pathways, weather conditions, public transportation and the lack of integral accessibility to services and systems. Although instrumental support could ease participation, it was also associated with an impaired sense of efficacy. Attitudes, particularly developing an understanding of the experiences of people with multiple sclerosis and gait impairment from others' perspectives, were considered important but often lacking, and discriminatory attitudes were experienced. On the level of personal factors, acceptance of both the mobility assistive devices and the disease itself were seen as facilitators for maintaining involvement in life situations.

**Conclusions:**

This study extends the existing literature by shedding light on the interconnectedness of contextual factors within the ICF and various facets of perceived participation, including a sense of connection, efficacy and meaning. These findings provide valuable insights for stakeholders such as urban planners and policymakers in developing inclusive environments that enhance the overall quality of participation for people with multiple sclerosis and gait impairment.

**Patient or Public Contribution:**

This study reports on the lived experiences of people with multiple sclerosis and gait impairment who (intend to) use mobility assistive devices. The research team stood in close exchange with project members of the German Multiple Sclerosis Society Lower Saxony, a group representing the interests of people with multiple sclerosis, to design and conduct the focus group interviews. The results from the focus group interviews were the basis for the design of a participatory future workshop in which people with multiple sclerosis and stakeholders involved in the healthcare process collaboratively developed recommendations for improving the provision of mobility assistive devices.

**Trial Registration:**

German Clinical Trials Register number: DRKS00025532.

## 1 Introduction

Approximately 2.5 million individuals worldwide are estimated to be affected by multiple sclerosis (MS) [1], making it the most prevalent neuroimmunological disorder [2]. MS presents with a range of symptoms, including motor function impairment and depression [3], which can impact the participation experiences of people with MS (PwMS).

The International Classification of Functioning, Disability and Health (ICF) [4] offers a framework that allows for classifying and analysing the biopsychosocial functioning of PwMS. It comprises two main parts: (a) components related to functioning and disability, and (b) components concerning contextual factors. Participation, defined in the ICF on p. 14 as ‘involvement in a life situation’ [4], represents one aspect of functioning and disability. It is coded alongside activities and encompasses the following domains: (1) learning and applying knowledge, (2) general tasks and demands, (3) communication, (4) mobility, (5) self‐care, (6) domestic life, (7) interpersonal interactions and relationships, (8) major life areas and (9) community, social and civic life. The contextual factors within the ICF delineate an individual's life context. They encompass personal and environmental factors and can function as barriers and facilitators to participation. Although personal factors are not further categorized within the ICF, environmental factors can be divided into the following: (1) support and relationships, (2) products and technology, (3) services, systems and policies, (4) natural environment and human‐made changes to environment and (5) attitudes. The ICF views disability as the result of an interplay between personal and environmental factors and an individual's health condition [4].

The lived experiences of PwMS with gait impairment are unique with regard to this interplay. Although being physically active is perceived as empowering by PwMS [5], it can be burdensome when gait impairment limits mobility and independence [6]. Balance problems impact the health‐related quality of life of PwMS [7], and mobility limitations are linked to reduced participation [8, 9] and a heightened risk of unemployment in PwMS [10]. Due to walking difficulties, PwMS often need to make adjustments to their work situations such as taking time off [11]. PwMS reported mobility limitations most frequently as the most disabling symptom of MS [8].

Numerous personal and environmental factors have been examined for their impact on the participation experiences of PwMS. As gait impairment is associated with the use of mobility assistive devices (MADs) in PwMS [12, 13], MADs, particularly manual wheelchairs, electric wheelchairs/scooters and walkers [13, 14], play a pivotal role in shaping experiences of being physically active [15] and participating in life [13]. They contribute to a sense of independence, adaptability, competence and self‐esteem [6, 13, 16, 17]. Souza et al. point out in their review that changes in functionality are often unpredictable, which can be challenging for both the PwMS and their social surrounding [13]. Maintaining social relationships is crucial for PwMS [18], but they often break apart [19]. Attitudes from the social surrounding and stigmatization [18, 20] impact the participation experiences of PwMS. Challenges with accessibility and the physical environment [21, 22] were also reported to shape participation experiences of PwMS. So far, it has not yet been examined how exactly MADs and other contextual factors interplay and thus shape the experience of a sense of efficacy, connection and meaning for PwMS with gait impairment.

When aiming at analysing participation experiences of PwMS with gait impairment who (intend to) use MADs, it is important to note that the concept of participation is multifaceted and subject to various theoretical perspectives. The relevance of considering not only objective aspects of participation but also the subjective experience of participation has been recognized [23]. This subjective experience of involvement [4] is not operationalized within the ICF. Several authors have highlighted this paucity within the ICF and emphasized the necessity of conceptualizing perceived participation within its framework [24, 25, 26, 27]. In our pursuit of gaining a comprehensive understanding of the participation experiences of PwMS with gait impairment, we will thus complement the ICF‐based perspective on participation in this study with its subjective facet: perceived participation. Previous studies have characterized perceived participation through various distinct facets [25, 26, 28]. The social identity approach (SIA) to health and well‐being [29] shares considerable common ground with these facets. This approach provides a theoretical foundation for understanding the subjective facets of participation. According to the SIA to health and well‐being [29], social identities stemming from relevant group memberships offer psychological resources, such as a sense of connection, efficacy and meaning, as well as effective social support. Depending on the nature and presence of these facets, health can be positively or negatively impacted. This approach has previously been employed in rehabilitation research [30] and served as a framework for analysing adjustment to living with MS [31]. The relevance of the facets described in the SIA to health and well‐being is substantiated by studies demonstrating that related themes hold significance in the lived experiences of PwMS [20, 32]. Therefore, perceived participation in this study will be characterized by the facets of a sense of connection, efficacy and meaning derived from the SIA to health and well‐being. Effective social support, categorized in the ICF as a contextual factor, will also be considered as such in this study. Given the framework provided by the ICF and the SIA to health and well‐being [29], this manuscript will explore the following research questions:

a. How do PwMS with gait impairment who (intend to) use MADs experience the ICF contextual factors, including support and relationships; products and technology; services, systems and policies; human‐made changes to environment and attitudes, and personal factors, in the context of their involvement in life situations?
b. How do they describe the interconnectedness of these contextual factors with their sense of connection, efficacy and meaning?

## 2 Materials and Methods

A multimethod qualitative research design was employed. Online focus group interviews were conducted, followed by online individual interviews with participants from the focus group interviews. This sequential approach allows for a comprehensive understanding of a range of experiences, followed by a deeper exploration through individual interviews [33]. The reporting of this study adheres to the Consolidated Criteria for Reporting Qualitative Research (COREQ) checklist [34], as outlined in Appendix S3. Ethical approval for this research was obtained from the corresponding research ethics committees (focus group interviews: Research Ethics Committee of the Hannover Medical School, reference number 9173_BO_K_2020; focus group interviews and individual interviews: Faculty of Health Sciences, University of Oldenburg, Germany, reference numbers 2020‐108 and 2021‐077).

### 2.1 Participant Selection and Setting

The purposive sampling strategy involved two stages: (1) recruitment for the focus group interviews and (2) recruitment for individual interviews from among the participants of the focus group interviews. Participants for the focus group interviews were recruited through the German Multiple Sclerosis Association Lower Saxony (DMSG Lower Saxony), a non‐profit organization advocating for the interests of PwMS [35]. Invitations, along with study information, an informed consent form and a demographic questionnaire, were dispatched to 433 members of the DMSG Lower Saxony. Those who agreed to participate sent their informed consent form to the research team at the University of Oldenburg.

Inclusion criteria were as follows: (a) individuals aged 18 years or older, (b) those who possessed a proficient command of the German language, (c) those who had received a diagnosis of MS and (d) those who either owned or were in the process of acquiring a walker, manual wheelchair or electric wheelchair/scooter. In the event of a higher number of requests than available slots in a focus group, participants were chosen with the aim of achieving a well‐balanced composition in terms of demographic and clinical factors. Out of the 29 PwMS interested in participating, one participant was unable to participate due to technical issues. Nine people did not fulfil inclusion criterion (d) (*n* = 6) or the intended group composition in the case of too many requests (*n* = 3). Consequently, a total of 19 PwMS were selected. As all 19 participants had consented to further contact for the potential inclusion in additional interviews, each of them was contacted during the second recruitment phase. Informed consent was obtained. Of the 18 participants successfully reached, 13 expressed their willingness to partake in an additional individual online interview.

### 2.2 Data Collection

The focus group interviews were conducted as part of ‘Multiple Sclerosis—Patient‐Oriented Care in Lower Saxony’ (MS‐PoV), funded by the German Federal Joint Committee's Innovation Fund (Grant Number 01VSF19046). The project MS‐PoV aims at analysing the healthcare provision of PwMS in Lower Saxony with a special focus on assistive devices. Questions in the focus groups revolved around factors influencing participation. In the semi‐structured individual interviews, the focus shifted towards the sense of connection, efficacy and meaning, and their relationship with contextual factors (see interview guide in Appendices S1 and S2). Both the individual and focus group interview guide underwent pilot testing with PwMS with gait impairment. Segments of the focus group interviews pertinent to this study were analyzed alongside the individual interviews.

Four focus group interviews, each consisting of four to six participants, and 13 individual interviews were conducted between May and August 2021 online, utilizing the videoconference tool BigBlueButton [36]. Technical information and information about the goals of the study were given during a precheck meeting conducted individually with participants. Participants were situated in their personal environments, utilizing electronic devices to participate in the online conferences.

The online focus group interviews were facilitated by R1, with support from R3. All individual online interviews were facilitated by R1. Additional members of the research group were present to facilitate session recording and provide technical support. The focus group interviews had a duration of approximately 1.5 h each. The subsection within each focus group interview relevant to this research study ranged from 20 to 32 min in length. The individual interviews' duration ranged from 18 to 56 min.

### 2.3 Data Analysis

All interviews were audio recorded and transcribed verbatim by a professional in compliance with the transcription guidelines established by Fuß and Karbach [37] (focus group interviews) or by a graduate student assistant with transcription rules based on Kuckartz's guidelines [38] (individual interviews). Identifying information was removed from the transcripts, and pseudonyms were assigned. One individual interview was excluded from the data analysis due to insufficient audio quality. In the final analysis, data from 19 PwMS with gait impairment who participated in one of the four focus group interviews and data from 12 participants who were also interviewed individually were analyzed. For data management and analysis, the software MAXQDA [39] was employed.

Two researchers with backgrounds in psychology (R1) and occupational therapy (R2) analyzed the data using qualitative content analysis as per Kuckartz [38] following a deductive–inductive approach. Deductive categories were established by referencing the definitions as described in the ICF [4] and the SIA to health and well‐being [29]. This initial deductive phase was followed by an inductive fine‐coding phase (Table 2). During the inductive development of subcategories, we permitted the incorporation of existing theoretical concepts to enhance theoretical coherence.

After initial textual analysis, the data set was divided into two parts. R1 and R2 independently coded the first part of the material (two focus groups and six individual interviews) using the main categories. Decision rules and anchor examples were documented in a codebook and further refined during the coding process. Discrepancies were addressed through iterative discussions until a consensus was reached. In cases of disagreement, R5 was consulted. Subsequently, R1 coded the second part of the material (two focus group interviews and six individual interviews), with R2 conducting checks of the coding and resolving any disagreements through discussion. Following this initial phase of structuring the material with the deductive coding system, the inductive fine‐coding phase commenced. Based on a part of the interview data, R1 and R2 generated independently suggestions for subcategories, which were discussed until consensus was achieved. The resulting fine‐coded category system was discussed with R5. The remaining focus group interviews and individual interviews from the second part of the data set were fine coded independently by R1 and R2. An intercoder reliability assessment for fine coding, yielding a *κ* score of 0.75 [40], was conducted. Because the coding demonstrated good intercoder reliability as per Kuckartz [38], R1 performed the fine coding for the remaining interviews from the first part of the data set, whereas R2 checked for any inconsistencies.

## 3 Results

Table 1 provides an overview of participant characteristics. Disability levels were assessed using the Expanded Disability Status Scale (EDSS), ranging from 0 (representing normal function) to 10 (indicating death due to MS), as outlined in Kurtzke [41], through self‐assessment [42]. Notably, EDDS values of 4 and above primarily indicate the extent of gait impairment [1].

**Table 1:** Sociodemographic and clinical characteristics of participants.

Characteristics	Focus group interviews	Individual interviews
Gender		
Female	12	6
Male	7	6
Age		
Mean (range)	53.6 (33–76)	54.8 (36–76)
Years since diagnosis		
Mean (range)	16.1 (2–33)	17.7 (8–33)
Form of MS		
RRMS	6	3
SPMS	6	4
PPMS	7	5
Disability (EDSS score)^a^		
Mild (≤ 3)	—	—
Moderate (4–5.5)	4	1
Severe (≥ 6)	15	11
EDSS score		
Median (range)^b^	6.5 (4–8)	6.75 (4–8)
Mobility assistive devices (MADs)		
Manual wheelchair	13	9
Electric wheelchair/scooter^c^	13	10
Walker	11	8
Other	6	5
Employment status		
Full‐time employment	3	2
Part‐time employment	3	3
Retirement pension	4	3
Disability pension	7	4
Other/not assignable	2	—
Highest general school certificate		
Lower secondary school diploma	1	—
Secondary school diploma	8	4
University‐entrance diploma/vocational diploma	9	8
Not assignable	1	—

*Note: N* = 19 for focus group interviews and *N* = 12 for individual interviews. All participants of the individual interviews also participated in the focus group interviews.

Abbreviations: EDSS, expanded disability status scale; PPMS, primary progressive multiple sclerosis; RRMS, relapsing‐remitting multiple sclerosis; SPMS, secondary progressive multiple sclerosis.

^a^ Classification followed Marrie et al. [43]; moderate disability indicates early gait impairment, whereas severe disability indicates assistive device for ambulation or being nonambulatory.

^b^ Three participants of the focus group/individual interviews marked two EDSS scores that lay right next to one another. One participant explained that both scores seemed adequate. The mean of the two scores was calculated and included in the calculation of the median and range.

^c^ Including electric power add‐on for manual wheelchairs.

Table 2 gives an overview of the category system. In total, 755 codes were assigned to the categories products and technology (289 codes); attitudes (115 codes); services, systems and policies (104 codes); support and relationships (90 codes); the natural environment and human‐made changes to environment (24 codes) and personal factors (133 codes). An additional fine analysis of the impact of the contextual factors on perceived participation is integrated in ‘Results’ section. Within this fine analysis, overlaps between contextual factor codes and codes within the categories of sense of connection, efficacy and meaning were analyzed. An analysis exclusively focusing on the 354 codes allocated to the categories of sense of connection, efficacy and meaning is planned to be published separately.

**Table 2:** Category system.

Main category^a^ (deductive development)	Definition of the main category	Subcategory^b^ (inductive development)
Support and relationships	Statements about the practical physical or emotional support, protection, nurturing, relationship and assistance given by people/animals that influence the degree or the perception of participation.	Evaluative support
		Emotional support
		Instrumental support
Products and technology	Statements about natural or human‐made products, equipment and technology existing in the immediate environment of an individual and that influence the degree or the perception of participation.	Mobility assistive device (MAD)
		Design of buildings
		Design of pathways
		Public transportation
		Everyday products
Services, systems and policies	Statements about (a) services that offer benefits, structured programmes and operations designed to meet the needs of individuals, (b) systems that are administrative control and organizational mechanisms, (c) policies constituted by rules, regulations, conventions and standards and that influence the degree or the perception of participation.	Integral accessibility
		Match of work structures
		Legal requirements
		Efforts of political actors
		Process of healthcare services
Natural environment and human‐made changes to environment	Statements about elements of the natural or physical environment, components of the environment that have been modified by people, as well as characteristics of human populations within that environment and that influence the degree or the perception of participation.	COVID‐19 pandemic
		Climate
Attitudes	Statements about attitudes that are observable consequences of customs, practices, ideologies, values, norms, factual beliefs and religious beliefs and that influence the degree or the perception of participation.	Perspective‐taking
		Being compassionate
		Openness
		Willingness to help
		Insecurity
		Discriminatory attitudes
Personal factors	Statements about aspects that relate to the individual and that influence the degree or the perception of participation.	Acceptance process
		Personality traits
		Knowledge
		Dealing with uncertainty
		Occupational qualification
Sense of connection	The sense of connection is defined as the subjective feeling of psychological closeness to other people and the feeling of being an integral part of a social system in the context of a participation domain.	—
Sense of efficacy	The sense of efficacy is defined as a subjective feeling of control or power by oneself in the context of a participation domain.	—
Sense of meaning	The sense of meaning encompasses a subjective feeling of significance and value in the context of a participation domain.	—

^a^ The definition of the main categories support and relationships; products and technology; services, systems and policies; natural environment and human‐made changes to environment; and attitudes and personal factors were based on the ICF (World Health Organization, pp. 171–207 for environmental factors and p. 214 for personal factors). The main categories, sense of connection, sense of efficacy and sense of meaning, were derived from the SIA to health and well‐being [29].

^b^ The subcategories of a sense of meaning, efficacy and connection derived from the SIA to health and well‐being [29] are not the focus of this article and will be published and discussed in a following publication.

The subsequent section presents the participation experiences described by the interviewees. The findings are organized according to the contextual factors of the ICF. Excerpts from individual interviews are marked with the abbreviation (II), whereas excerpts from focus group interviews are marked with the abbreviation (FG).

### 3.1 Support and Relationships

Instrumental support, encompassing tangible actions that influence individuals' involvement in various life situations, played a major role in the lives of the interviewees. In addition to assuming responsibilities in tasks such as household chores, work‐related tasks or self‐care, these supportive actions also centred on interactions with the MAD, such as assisting with navigation within a building or pushing a wheelchair. These mobility‐related supportive actions eased the involvement in life situations, such as leisure activities. Although two interviewees mentioned that instrumental support elicited a sense of connection, the overall experience of needing and receiving instrumental support primarily influenced the level of self‐efficacy, often intensifying feelings of dependence that were perceived as burdensome. By exploring the role of instrumental support from his wife, one participant elaborates on the impact the instrumental support has on his sense of independence, stating, ‘[…] it is a burden to me […] when […] my wife is helping me again and again. […] She's then always responsible: yes I help, I help! That is all well and good but each time I realize again: You can not do it, you can't do it! That's getting me down. Really Down’ (II 1).

Emotional support is directed at an enhanced emotional processing of experiences in participation domains. It is frequently described as ‘stand by’ (II 2). It emerged as a buffering factor in difficult times during the course of MS, for example at the time of diagnosis or when experiencing discrimination. It was deemed essential and described as a ‘present’ (II 2) or ‘giving strength’ (II 3). The experience of emotional support was accompanied by a sense of connection in terms of experiencing a sense of inclusion in a participation domain.

Furthermore, interviewees also mentioned actions that reflect evaluative support, which aims at the adaptation of the interviewees' appraisal of a situation. By engaging in a joint reevaluation of the prospects for sustained participation in life situations and joint reevaluation of the role of a MAD in their lives, this form of support was described as an enabler of adapting to life with MS and using a MAD.

### 3.2 Products and Technology

The MADs played a major role in the lives of the interviewees. Although various MADs, including walking sticks and foot lifters, were discussed, the interviews primarily centred around walkers, manual wheelchairs and electric wheelchairs or scooters. These MADs serve distinct functions with respect to participation experiences.

Specifically through the creation of mobility, the interviewees describe the MADs as enablers for participation in various aspects of life, including work, leisure activities and accessing healthcare services as illustrated here: ‘It is completely clear, without my walker or my scooter or my wheelchair, I could not participate in anything’ (FG 2). MADs also functioned as facilitators, not merely determining the feasibility of participation but influencing the overall experience of involvement. This facilitation often accompanied a sense of comfort and ease, as articulated in the following quote: ‘[…] when I am in a wheelchair, I am personally more relaxed, I do not need to worry […] this facilitates many things […]’ (FG 1). Some participants highlighted the importance of being able to switch flexibly between different MADs based on the specific demands of a situation as a prerequisite for enabling or facilitating their participation.

Although the utilization of MADs introduced new challenges, such as the handling of these devices, they were predominantly valued. A central experience of the interviewees was that MADs promoted a sense of efficacy by enabling the experience of a sense of independence and a sense of competence in participation domains: *‘*[…] for the independence, the electric wheelchair is invaluable’ (II 4). This is also reflected in the finding that MADs functioned as a substitution or reduction of the need for instrumental support. Despite the high level of appreciation for MADs, some interviewees expressed awareness of a new form of dependence on these devices.

With regard to a sense of connection, MADs were noted for creating a sense of sameness in situations where the MAD served as a social link, such as during wheelchair sports. They were also recognized as important gait‐keepers for doing meaningful and joint activities with other significant people where a sense of inclusion could be experienced. In contrast, the interviewees also expressed a sense of otherness due to the distinctiveness arising from their MADs, particularly when feeling to differ from others due to the MAD. The MAD also functions as a determinant for position in social interactions, which was described by one participant.

The functioning of the MADs and the overall quality of participation depend largely on other products and technology, such as the design of pathways, design of buildings and public transportation. Public transportation, particularly buses and trains, was perceived as inadequately accessible, with a lack of functioning lifts and entrances being a common issue. This often rendered the use of public transportation difficult, if not impossible and unappealing. For smooth mobility, well‐designed pathways were considered essential for users of electric wheelchairs/scooters, manual wheelchairs or walkers. However, various obstacles, such as the absence of lowered curbs, cobblestones or uneven sidewalks, were reported.

In the context of building design, non‐barrier‐free entrances with heavy doors, a lack of door openers, stairs and narrow hallways were highlighted as complications that hindered participation in activities such as work, shopping or dining at a restaurant. These difficulties resulted in a diminished sense of efficacy when feeling helpless and dependent, as expressed by one participant: ‘In a building without an elevator but with stairs […] it gets very difficult [.] So your disability is being—you are disabled—but your disability is being made, the access is basically denied’ (FG 1). Some participants point out that these challenges were so exhausting that they ultimately refrained from engaging in certain activities.

Concerning building design, the presence of ramps, lifts and barrier‐free bathrooms was deemed important for facilitating participation. However, they were sometimes out of reach and thus inaccessible in buildings, incompatible with MADs or unavailable. Although a barrier‐free building design could make participation in life situations possible, it did not necessarily guarantee the quality of participation. To illustrate, a participant described their experience attending a theatre show in a building with designated wheelchair places, yet subjectively, the quality of participation, in terms of a sense of connection, was compromised. This resulted in a feeling of exclusion: ‘There are extra wheelchair places with only wheelchair users and right there you spend your time during the performance. It is unpleasant because you don't sit next to your partner […] you enjoy the performance more or less alone.’ and he summarizes: ‘[…] when you are there […] on your own—you feel lonely. I feel lonely’ (II 7).

### 3.3 Services, Systems and Policies

Integral accessibility encompasses accessibility as a pervasive feature across systems and services, and it has often been perceived as lacking, especially in the area of health services, transport services and urban planning. Determining whether a service or system offers integral accessibility has proven challenging.

This challenge extends to the match of work structures. Specifically, participants noted that when adjustments to working conditions were implemented, such as shift changes or accommodating work in accordance with MS symptoms, PwMS with gait impairment were able to maintain their work to a certain extent. However, when the nature of the work did not allow for such adaptations, continuous employment was reported to be problematic.

Legal requirements are frequently seen as obstacles to creating an environment conducive to the participation of PwMS with gait impairment as exemplified here: ‘Why can't I get a disabled parking permit […]? Because I only have a walker. […] There are requirements for any stuff’ (FG 3). Political actors taking proactive steps to address the needs of PwMS were deemed crucial but currently lacking.

The process of healthcare services, particularly the delivery process of MADs and access to an assistant can facilitate participation in various life domains. Conversely, receiving advice from a neurologist discouraging the use of a MAD was cited as a hindrance to achieving a high quality of participation.

### 3.4 Natural Environment and Human‐Made Changes to Environment

The measures put in place to contain the pandemic restricted the ability to be involved in various life situations. Some interviewees reported that their social gathering places were located in healthcare service settings, such as nursing homes or rehabilitation centres. In these cases, digital everyday products that enabled Internet usage were mentioned as valuable tools for maintaining contact with others.

Climate was identified as another factor affecting participation by some interviewees. Feeling not self‐efficient in using the MAD during adverse weather conditions was described as burdensome and also led to refraining from outdoor activities. Also, due to a lack of wheelchair‐accessible facilities, visiting a café was restricted to the summer when outdoor seating was available.

### 3.5 Attitudes

Attitudes were experienced in various dimensions. One aspect reported was perspective‐taking, which involves a person's willingness to place themselves in the position of a PwMS, develop an understanding of the experiences of PwMS with gait impairment and subsequently consider PwMS needs. This issue often centred on a lack of comprehension regarding the impact of MS‐related symptoms on an individual's participation in various life situations. Lack of perspective‐taking was primarily encountered within the broader society; however, it could also manifest in interactions with immediate family members or medical professionals. Some interviewees particularly noticed this issue in situations where they felt a diminished sense of efficacy, especially when they felt helpless. Effective perspective‐taking, whether by colleagues, supervisors, medical professionals or other PwMS, often led to actions taken by these individuals to facilitate participation in life situations, as exemplified here: ‘[…] the colleagues now always take care that I have free passage for my electromobile at work […]’ (II 5).

In contrast to perspective‐taking, the attitude of openness involves a goodwill, but may not necessarily include the interviewees' emphasis on developing an understanding of the experiences of PwMS. Although there were also instances of lacking openness, the prevailing experience was one of openness, particularly in relation to colleagues, employers, friends, children and medical professionals. Interviewees noted that employee openness was often linked to an alignment with work structures and was associated with a heightened sense of connection, including a sense of intimacy and inclusion.

Two additional facets of attitudes were reported: willingness to help and being compassionate. Willingness to help was evident in interactions with immediate family members, in the workplace, in public and during leisure activities, leading to the availability of instrumental support. Compassion from others regarding the life situation and feelings of PwMS was described as a two‐sided experience. When it leaned towards shared joy, compassion was considered desirable. However, when this compassion was associated with pain and impairment, it was perceived as burdensome, as expressed by one interviewee: ‘And I have to say the pitying looks when one limps along heavily with a pain contorted face, that is not nice to see’ (II 6).

PwMS with gait impairment also reported experiencing the perception of insecurity from others regarding how to interact with them and encountering discriminatory attitudes. These attitudes manifested in demeaning actions from the general population, such as improperly using disabled parking spaces and issuing verbal threats when confronted about such behaviour.

### 3.6 Personal Factors

The acceptance process of living with MAD and MS was a frequent topic of discussion among the interviewees. Acceptance was viewed as dynamic and could vary widely. Initial non‐acceptance was in some cases accompanied by efforts to conceal symptoms and a reluctance to use a MAD. Acceptance was marked by, first, recognizing that certain activities could no longer be performed in the same manner as before, and second, understanding that acceptance could pave the way for the rediscovery of meaningful participation in various domains.

Several factors were identified as playing a role in initiating the acceptance process. These included experiencing difficulties in participating in daily life without a MAD, time for processing and encountering key moments that had a profound impact on their perspective.

To illustrate the influence of a key moment on the acceptance process, one interviewee shared a personal experience involving a bet at a shopping centre: ‘The woman said: why don't you take a wheelchair? […] I said: Madam, I am not going to sit in a wheelchair (.) and then we made a bet [about whether the wheelchair is a facilitation]. It was a great facilitation […] I said: Shit you have won. That's how I got to get the wheelchair’ (II 7).

In this anecdote, the interviewee's encounter with a challenging situation led to a change in perspective and ultimately contributed to their acceptance of using a wheelchair.

In addition to personality traits such as taking initiative and optimism, the interviewees highlighted the significance of professional qualifications and knowledge. Furthermore, they noted that information regarding the accessibility of public places was often insufficient or required excessive effort to gather. Consequently, they frequently found themselves grappling with uncertainties when attempting to participate in various aspects of life. When the level of uncertainty became overwhelming, or the planning process became overly burdensome, some participants chose to refrain from certain activities.

## 4 Discussion

The goal of this research article was twofold: to analyse environmental and personal factors perceived as relevant by PwMS with gait impairment who (intend to) use a MAD for their involvement in life situations and to investigate how these factors shape the experience of a sense of connection, efficacy and meaning.

With their role as facilitators and enablers for the individuals' involvement in life situations, the MADs reduced the need for instrumental support while increasing a sense of efficacy. These findings corroborate earlier research results that underscore the importance of MADs in fostering independence for PwMS and those with mobility impairments [13, 44, 45]. However, the utilization of MADs also introduced new challenges and dependencies related to navigating them. For example, accessible facilities for self‐help groups were significantly affected by a lack of closure during the COVID‐19 pandemic [46], potentially rendering PwMS with gait impairment, who relied on MAD‐accessible facilities, especially vulnerable to pandemic‐related restrictions.

The interviews also reveal a multifaceted interplay between MADs and other products and technologies. In social groups where all group members use MADs, such as in wheelchair sports, the MAD symbolizes similarity and consequently fosters a sense of connection. Bates et al. [47] specifically investigated the role of wheelchair basketball in a young individual's well‐being. The authors describe that through wheelchair basketball, a place is created, which is specifically designated to wheelchair users. Consequently, social inclusion and participation can be experienced. However, the results also indicate that conventional methods that aim at facilitating participation for people using MADs, such as designated seating positions for wheelchair users in theatres, were reported to diminish the sense of connection. Given that the perception of belonging is a central component of inclusion [48], it can be inferred that in such cases, full inclusion is then not realized. The results reveal challenges in creating an inclusive environment for users of MADs and the relevance of context that decides which experiences and senses arise. It underscores the importance of considering the subjective experiences of participation when creating accessibility solutions that aim at effectively fostering inclusion.

Although instrumental support, frequently in relation to handling the MAD, facilitated participation, the experience of relying on such support was linked to a diminished sense of efficacy. This ambivalent role of instrumental support is reflected in the results of previous studies: although Kleiboer et al. demonstrated that an excess of instrumental support within a partnership can negatively affect the self‐esteem of PwMS [49], Eizaguirre et al. found instrumental support to be a predictor for health‐related quality of life [50]. Future studies could examine the influence of instrumental support on various dimensions of participation experiences, including the sense of efficacy.

Similar to the barriers identified by Green and Todd [20], this interview study also revealed instances of discriminatory attitudes and inadequate perspective‐taking in relation to the MAD. In this research, attitudes were frequently attributed to the general population without specifying a particular source. Coenen et al. [21] similarly discovered in their study on contextual factors influencing participation among PwMS in Germany that societal attitudes were frequently cited by participants, alongside attitudes from other stakeholders. Conversely, Jalili et al. [51] and Hamed [52] found in their studies that attitudes at home and those of first‐degree family members were the most significant for PwMS. Importantly, Jalili et al. [51] emphasized that their results, along with those of Hamed [52], could be influenced by the cultural context of the population studied, which prioritized family‐centred values. The findings presented here extend discussions regarding potential differences in the nature and sources of attitudes and their impact on the participation experiences of PwMS across cultures, particularly in relation to the degree of family‐centredness, as elucidated by Jalili et al. [51]. Future studies may consider this as a foundation for further exploration.

On the level of personal factors, this study highlighted the highly individualized nature of accepting both MS and MADs. This finding resonates with the outcomes observed by Squires et al. [45], indicating that, within this population, accepting MS and accepting MADs were distinct yet interrelated experiences. The significance of accepting MS and MADs has been previously underscored in the context of living with MS [21, 45].

Integrating the ICF contextual factors with the SIA to health and well‐being [29] can enhance our comprehension of how the context interacts with participation experiences on an emotional level. This approach presents an innovative method for examining the relationship between an individual's context and the participation quality through a theory‐driven perspective.

The participants of this study cover different facets of the sociodemographic and clinical parameters, including gender, age, level of disability, level of education, employment status, sort of MAD used or intended to use, disease duration and form of MS. As gait impairment was a central aspect of this study, the trend towards higher EDSS scores tends to be understandable, as gait impairment is a central characteristic of higher EDSS scores [41].

There are some limitations to this study. First, the primary focus of the study was on the MADs. Studies that do not place as much emphasis on the role of MAD may yield different focus areas and subcategories in relation to contextual factors. Furthermore, we chose to utilize only the first‐level contextual factor categories of the ICF to allow for the exploration of MS‐specific experiences. Although this approach may limit the comparability of our results with studies that comprehensively code material using all ICF categories, it was intended to uncover aspects specifically relevant from the perspective of PwMS with gait impairment who (intend to) use MADs. Lastly, the research design is qualitative, limiting the insights gained from this study to the subjective experiences of the PwMS interviewed in this study. Future quantitative studies could follow up on the findings of this study.

## 5 Conclusions

By ratifying the Convention on the Rights of Persons with Disabilities, Germany acknowledges the rights of individuals with disabilities to participate in various aspects of life on an equal basis. This study suggests that PwMS with gait impairment who (intend to) use MADs encounter challenges associated with the interaction between various contextual factors, which impact their sense of connection, efficacy and meaning. As the global prevalence of MS continues to rise [53], there is a growing societal imperative to implement measures that guarantee a high quality of participation for PwMS experiencing gait impairments. Stakeholders, including urban planners, political decision‐makers and scientific researchers, can harness these insights to promote the creation of an inclusive environment.

## Author Contributions

**Elise‐Marie Dilger:** conceptualization, methodology, investigation, formal analysis, writing–original draft, writing–review and editing. **Nadja Reeck:** formal analysis, writing–review and editing. **Dyon Hoekstra:** investigation, writing–review and editing. **Annett Thiele:** writing–review and editing. **Anna Levke Brütt:** conceptualization, methodology, supervision, formal analysis, writing–original draft, writing–review and editing.

## Disclosure

During the conduct of the study and when this paper was written, four out of the five authors self‐identified as female and one author self‐identified as male. For protecting the identity of the participants, the transcripts of the focus group and individual interviews are not publicly accessible. Further anonymized excerpts for specific categories can be obtained by contacting the first author and upon giving a comprehensible rationale.

## Ethics Statement

Ethical approval for this research was obtained from two distinct research ethics committees: the Research Ethics Committee of Hannover Medical School (reference number 9173_BO_K_2020) for the focus group interviews and the Research Ethics Committee of the Faculty of Health Sciences at the University of Oldenburg, Germany (reference numbers 2020‐108 and 2021‐077) for both the focus group interviews and individual interviews. Written informed consent for participating in the study and for the publication of the study was obtained from the participants.

## Conflicts of Interest

The authors declare no conflicts of interest.

## Supporting information

Supporting information.

Supporting information.

Supporting information.

## Data Availability

For protecting the identity of the participants, the transcripts of the focus group and individual interviews are not publicly accessible. Further anonymized excerpts for specific categories can be obtained by contacting the first author and by giving a comprehensible rationale.
