# Supplementary material for: Impact of Contextual Factors on the Perceived Participation of People With Multiple Sclerosis and Gait Impairment Using Mobility Assistive Devices: A Qualitative Analysis
Source: Health Expect. 2024 Sep 28;27(5):e70033. doi: 10.1111/hex.70033 (PMC11437357; doi:10.1111/hex.70033)
Supplement: Supplementary file 1 — Supporting information. [file HEX-27-e70033-s002.docx]

**Interview guide for focus groups with People with Multiple Sclerosis (MS)**

**Focus: assistive device provision process:**

1. **There are many steps before the assistive device is in your hands and fits well. What experiences have you had in this process?**

*[if a specific 'phase' is described or mentioned in the discussion - then continue with the corresponding focus block for that phase (1.1 – 1.10)]*

1. How did the need to get an assistive device arise?
2. How did the idea of getting an assistive device come about?
3. How was it determined which assistive device to get?
4. What can you tell about the financing and/or about the approval process with the health insurance fund/other payers?
5. How satisfied are you with the delivery of the assistive device?
6. After receiving the assistive device, what happens next?
7. Did it happen that you wanted to receive an assistive device but did not get it? If so, what was the reason?
8. What was the reason for the failure of the provision process with the assistive device?

**Focus: Impact of assistive devices**

*[The questions of this section were specifically developed for the present study. The data generated with these questions was extracted from the transcripts and analyzed together with the data from the individual interviews.]*

**2. What can you do well again with the assistive device?**

1. What social or community activities can you participate in well again with the assistive device?
2. What is the impact of the assistive device on your daily life at work?

**3. Who or what makes it difficult for you to carry out the activities or participate in the areas of your life that are important to you?**

1. What difficulties in the activities and participation in the areas of life that are important to you are perhaps also experienced because of the assistive device?

**4. Who or what facilitates the activities and participation in the areas of life that are important to you?**

**5. Imagine you were talking to someone who does not have MS and does not use an assistive device. How would you describe to the person the importance of the assistive device to the quality of life and wellbeing of people with MS?**

**6. In contrast, in what areas of your life do you not use your assistive device?**

1. What is different about these situations compared to the situations where you use it?

**Focus: Suggestions for improvement**

**7. What do you think needs to change to improve the provision of assistive devices for people with MS?**

**8. Do you have any other important comments or additions regarding the provision of assistive devices for people with MS that we have not yet addressed in this focus group?**

**9. We have talked a lot today about the walker, the manual wheelchair and the electric wheelchair/scooter. Which assistive devices do you think should also be explored in more depth?**

**10. Which stakeholders of the provision process of assistive devices do you think we should invite to future focus groups?**

*[The order of the questions was flexible depending on the course of the focus group discussion]*
