# Supplementary material for: Impact of Contextual Factors on the Perceived Participation of People With Multiple Sclerosis and Gait Impairment Using Mobility Assistive Devices: A Qualitative Analysis
Source: Health Expect. 2024 Sep 28;27(5):e70033. doi: 10.1111/hex.70033 (PMC11437357; doi:10.1111/hex.70033)
Supplement: Supplementary file 2 — Supporting information. [file HEX-27-e70033-s003.docx]

**Interview guide for individual interviews with People with Multiple Sclerosis (MS)**

*Abbreviations:*

pd = participation domain

feel = feeling

sg = social group

**Focus: Identifying participation domains that are important for the participant**

1. **At the end of the focus group interviews, you have discussed with the other participants what you can do again with the assistive device but also which difficulties exist when using the assistive device in everyday life. At the beginning, together with you, I would like take a step back. First of all, regardless of the assistive device, what would you say, what are the life domains and activities that are important for you? In what areas of life is it important for you personally to be involved and to take part?**
   1. What would you miss if you could not do it anymore?
2. **You have described areas in your life that are important to you. Which assistive devices do you use in these areas of life?**
   1. Are there areas in which you use the assistive devices more often?

**Focus: In depths discussion of experiences in the respective participation domain**

*[the following section can be conducted repeatedly with each participation domain that was mentioned in question 1.]*

1. **I would like to firstly exchange about *pd*. How would you describe it, what is it that makes *pd* important for you?**
   1. What people or activities make this life area meaningful for you?
   2. What do you feel, when being involved in *pd*?
      1. Can you describe a specific situation when you felt especially *feel*?
         1. What strengthens those feelings?
         2. When do you not have those feelings?
2. **How would you describe the influence of you MS on *pd*?**
3. **How do you experience *pd* with your assistive device?**
   1. How did the involvement in *pd* change since having the assistive device?
      1. How does the assistive device influence the interaction?
         1. And how does this impact your feelings?
   2. What difficulties do you experience in *pd* with your assistive device?
      1. How does it make you feel?
4. **How much do you feel like being a part of *sg*?**
   1. Can you describe a specific situation in which you felt especially like you belong?
   2. What strengthens the feeling of being a part of *sg*?
   3. When do you have a particularly strong feeling of being part of *sg*?
   4. What impairs the feeling of being part of *sg*?
   5. When do you not have this feeling?
   6. How does the assistive device influence the feeling of being a part of *sg*?
5. **How would you describe your individual influence and your contribution to shaping the *pd*?**
   1. What does this mean for you?
   2. Can you describe a specific situation in which you felt influential?
      1. What strengthens the feeling of being influential?
      2. When do you feel especially influential?
      3. What impairs the feeling of being influential?
      4. When do you not have this feeling?
   3. How does the assistive device help you in shaping the *pd*?
      1. How did your experience of influence in the *pd* change since having the assistive device?
6. **If you could no longer be involved in *pd*, what would that mean to you?**
   1. What is it in the *pd* that gives you strengths?
   2. What is it in the *pd* that takes away strength from you?
   3. When does the assistive device give you strengths?
   4. When does the assistive takes away strength from you?

*[The order of the questions was flexible depending on the course of the individual interview]*
